# A new method for inferring timetrees from temporally sampled molecular sequences

**DOI:** 10.1371/journal.pcbi.1007046

**Published:** 2020-01-17

**Authors:** Sayaka Miura, Koichiro Tamura, Qiqing Tao, Louise A. Huuki, Sergei L. Kosakovsky Pond, Jessica Priest, Jiamin Deng, Sudhir Kumar

**Affiliations:** 1 Institute for Genomics and Evolutionary Medicine, Temple University, Philadelphia, Pennsylvania, United States of America; 2 Department of Biology, Temple University, Philadelphia, Pennsylvania, United States of America; 3 Department of Biological Sciences, Tokyo Metropolitan University, Tokyo, Japan; 4 Research Center for Genomics and Bioinformatics, Tokyo Metropolitan University, Tokyo, Japan; 5 Center for Excellence in Genome Medicine and Research, King Abdulaziz University, Jeddah, Saudi Arabia; University of New South Wales, AUSTRALIA

## Abstract

Pathogen timetrees are phylogenies scaled to time. They reveal the temporal history of a pathogen spread through the populations as captured in the evolutionary history of strains. These timetrees are inferred by using molecular sequences of pathogenic strains sampled at different times. That is, temporally sampled sequences enable the inference of sequence divergence times. Here, we present a new approach (RelTime with Dated Tips [RTDT]) to estimating pathogen timetrees based on a relative rate framework underlying the RelTime approach that is algebraic in nature and distinct from all other current methods. RTDT does not require many of the priors demanded by Bayesian approaches, and it has light computing requirements. In analyses of an extensive collection of computer-simulated datasets, we found the accuracy of RTDT time estimates and the coverage probabilities of their confidence intervals (CIs) to be excellent. In analyses of empirical datasets, RTDT produced dates that were similar to those reported in the literature. In comparative benchmarking with Bayesian and non-Bayesian methods (LSD, TreeTime, and treedater), we found that no method performed the best in every scenario. So, we provide a brief guideline for users to select the most appropriate method in empirical data analysis. RTDT is implemented for use via a graphical user interface and in high-throughput settings in the newest release of cross-platform MEGA X software, freely available from http://www.megasoftware.net.

## Author summary

Pathogen timetrees trace the origins and evolutionary histories of strains in populations, hosts, and outbreaks. The tips of these molecular phylogenies often contain sampling time information because the sequences were generally obtained at different times during the disease outbreaks and propagation. We have developed a new method for inferring divergence times and confidence intervals for phylogenies with tip dates. The new Relative Times with Dated Tips (RTDT) methods showed excellent performance in the analysis of computer-simulated datasets, producing similar or better results in several evolutionary scenarios as compared to other fast, non-Bayesian methods. The new method is available in the cross-platform MEGA software package (version 10.1 and higher) that provides a graphical user interface and allows usage via a command line in scripting and high throughput analysis (www.megasoftware.net).

## Introduction

Molecular phylogenetics enables the dating of the origin of pathogens and the emergence of new strains [1–3]. Typically, strains are sampled from individuals and populations during an ongoing or historical outbreak [4–9]. When sequences are paired with their sampling times, it becomes possible to calibrate molecular phylogenies of pathogen sequences and infer the timing of pathogen evolution. For example, HIV-1 sequences have been sampled at various times and geographic locations following its initial characterization in 1983 [2, 9, 10]. Analyses of sequences extracted from circulating strains and “archived” strains from preserved tissue samples have established that HIV-1 (group M) entered the human populations in the early 20^th^ century in Sub-Saharan Africa [10] and that subsequently dispersed across the globe [11, 12].

Many competing methods are available to build pathogen timetrees that estimate the timing of divergence of lineages in the tree [13–22]. In these analyses, the tips in a phylogeny are non-contemporaneous, and sampling times serve as calibrations that provide a means to date historical sequence divergences. These analyses are different from those used for the estimation of species divergence times because the sampling times of sequences from different species are effectively simultaneous. The difference in the sampling years for all sequences in interspecies datasets can be assumed to be effectively zero when compared to the time-scale of speciation.

The Bayesian framework underlies many of the widely-used tools for building pathogen timetrees (MCMCTree [15] and BEAST [14]). The use of Bayesian methods requires researchers to specify a clock prior that governs the change of evolutionary rate over lineages and a coalescent model or a speciation model (e.g., birth-death process) to generate a tree prior [14, 15]. Such information is rarely available *a priori*, and time estimates can vary when using different priors [23], resulting in alternative biological interpretations [15, 24]. Meanwhile, Bayesian methods often require long computational times, which makes them infeasible for analyzing datasets with thousands of sequences in contemporary molecular epidemiology [16, 19, 22].

Here, we present an approach based on the relative rate framework underlying the RelTime method [25, 26]. The RelTime method is attractive because it is not computationally demanding, and it does not require explicit clock and coalescent model priors. Both simulated and empirical analyses have shown RelTime to perform well for dating species evolution [25–27]. The new approach advances RelTime by relaxing the requirement that all tips in the phylogenetic tree are contemporaneous (i.e., sampling time *t* = 0), making it suitable for dating of pathogenic strains. We call it the RelTime with Dated Tips (RTDT) approach. Similar to RelTime, RTDT is an algebraic approach, so it is lightning fast and distinct from other approaches. For example, TreeTime [19] is a maximum likelihood approach that uses a normal prior to control the rate variation to make the clock to be more autocorrelated-like or more independent-like, and it implements a skyline coalescence model. Least Squares Dating (LSD) [16] uses least-squares criteria, and treedater [22] uses likelihood and least-squares jointly. LSD assumes the rate noise to be independent among branches within its clock framework, and treedater assumes branch rates to vary independently. In contrast, RTDT is based on an algebraic relative rate framework and does not make any explicit assumptions about evolutionary rate autocorrelation and independence varying.

Through the analysis of simulated datasets generated under different assumptions and empirically derived phylogenies, we compared the accuracy of dates and confidence intervals (CIs) estimated by RTDT with those produced by software implementing Bayesian methods (BEAST [14] and MCMCTree [15]) and non-Bayesian approaches (Least Squares Dating, LSD [16], TreeTime [19], and treedater [22]). These comparisons are more extensive than ever reported before, as our analyses involved the largest number of methods ever tested and the most extensive collection of simulated datasets and different rate variation scenarios explored. Furthermore, in the past, studies of benchmarking these methods have generally reported the accuracy of estimation of substitution rates or the age of the root node of phylogeny [13, 19, 20, 22]. To et al. [16] reported the average of the absolute and relative differences in actual and estimated times for all the nodes in simulated analysis to compare methods. However, this measure does not detect node-specific biases and patterns.

Therefore, the accuracy of node-by-node age estimates remains to be evaluated, which we have reported here. Also, previous studies have only used simulated computer datasets in which the independent branch rate (IBR) model was applied. In addition to datasets simulated under IBR model, we report the performance of all methods for phylogenies in which branch rates were autocorrelated (ABR model). This is important because HIV-1 subtype F, HIV-1 subtype D, HIV-2, and influenza phylogenies showed highly significant autocorrelation of rates (**Table 1**). In fact, MCMCTree provides an ABR model for tip-dating, and TreeTime implicitly employs rate correlation, but their performances have not been tested by using datasets that have evolved with ABR. Therefore, our analyses produce an extensive assessment of the performance of divergence time estimation by using available Bayesian and non-Bayesian methods.

**Table 1:** Empirical datasets used in this study.

		Time Estimates (year)		Clock model	
Virus	Node*	RTDT	Reported in the Reference	CorrTest	Reference
*HIV-1 Subtype F (154 sequences*, *1293 bps)*^*a*^				Autocorrelated	Mehta, et al.
	Node 1	1985.3 (1980–1987)	1980 (1975–1985)		(2011)
	Node 2	1985.1 (1980–1988)	1978 (1972–1983)		
	Node 3	1980.0 (1977–1982)	1973 (1966–1980)		
*HIV-1 Subtype D (24 sequences*, *2173 bps)*^*a*^				Autocorrelated	Parczewski, et al.
	Node 1	2003 (1999–2005)	2001 (1999–2005)		(2012)
	Node 2	2000 (1991–2003)	1999 (1992–2001)		
	Node 3	1995 (1984–1997)	1997 (1994–1998)		
	Node 4	2006 (1998–2007)	2003 (1999–2005)		
*HIV-1 Subtypes B/D (38–133 sequence*, *1497–8877 bps)*^*a*^^,^^*d*^				Mixed^x^	Worobey, et al.
	Node 1	1960–1966 (1948–1971)	1966–1969 (1961–1972)		(2016)
	Node 2	1963–1969 (1945–1974)	1969–1972 (1966–1974)		
	Node 3	1967–1970 (1949–1975)	1969–1974 (1967–1975)		
*HIV-2 (33 sequences*, *1107 bps)*^*b*^				Autocorrelated	Stadler and Yang
	Node 1	1983 (1978–1985)	1938–1941 (1952–1973)		(2013)
	Node 2	1985 (1979–1985)	1956 (1922–1957)		
	Node 3	1985 (1975–1986)	1961–1964 (1944–1966)		
*Rabies (67 sequences*, *1350 bps)*^*a*^				Independent	McElhinney, et al.
	Node 1	1967 (1936–1971)	1885 (1848–1914)		(2011)
	Node 2	1971 (1936–1972)	1917 (1894–1937)		
	Node 3	1982 (1936–1973)	1931 (1914–1947)		
	Node 4	1973 (1936–1973)	1941 (1925–1955)		
*Influenza A (289 sequences*, *1710 bps)*^*c*^				Autocorrelated	Stadler and Yang
	Node 1	1912 (1898–1916)	1813–1910 (1760–1917)		(2013)
	Node 2	1915 (1898–1918)	1832–1914 (1787–1918)		
	Node 3	1928 (1910–1930)	1889–1926 (1857–1929)		

a: BEAST with lognormal rates

b: MCMCtree with constant and autocorrelated clock models

c: BEAST with lognormal rates and MCMCtree with constant, independent, and autocorrelated clock models.

The range of estimated times based on these different methods was given.

d: The range of time estimates was obtained based on eight different subdatasets.

x: Five datasets showed autocorrelated rates and three independent rates.

*: Node IDs were given in **Figs** 2 and 5**A** and **S2 Fig**.

Here, we first present the algorithm for the new method, RTDT. We then evaluate the node-by-node accuracy of dates and CIs estimated by RTDT together with Bayesian (BEAST and MCMCTree) and non-Bayesian (LSD, TreeTime, and treedater) methods using simulated datasets. This evaluation of different methods yielded new insights into the performance of tip-dating methods in building pathogen timetrees, which formed the basis of our brief guidelines for researchers to select the best method for their dataset.

## Results

### New approach (RTDT) for estimating divergence times using temporally sampled sequences

**Fig 1:**
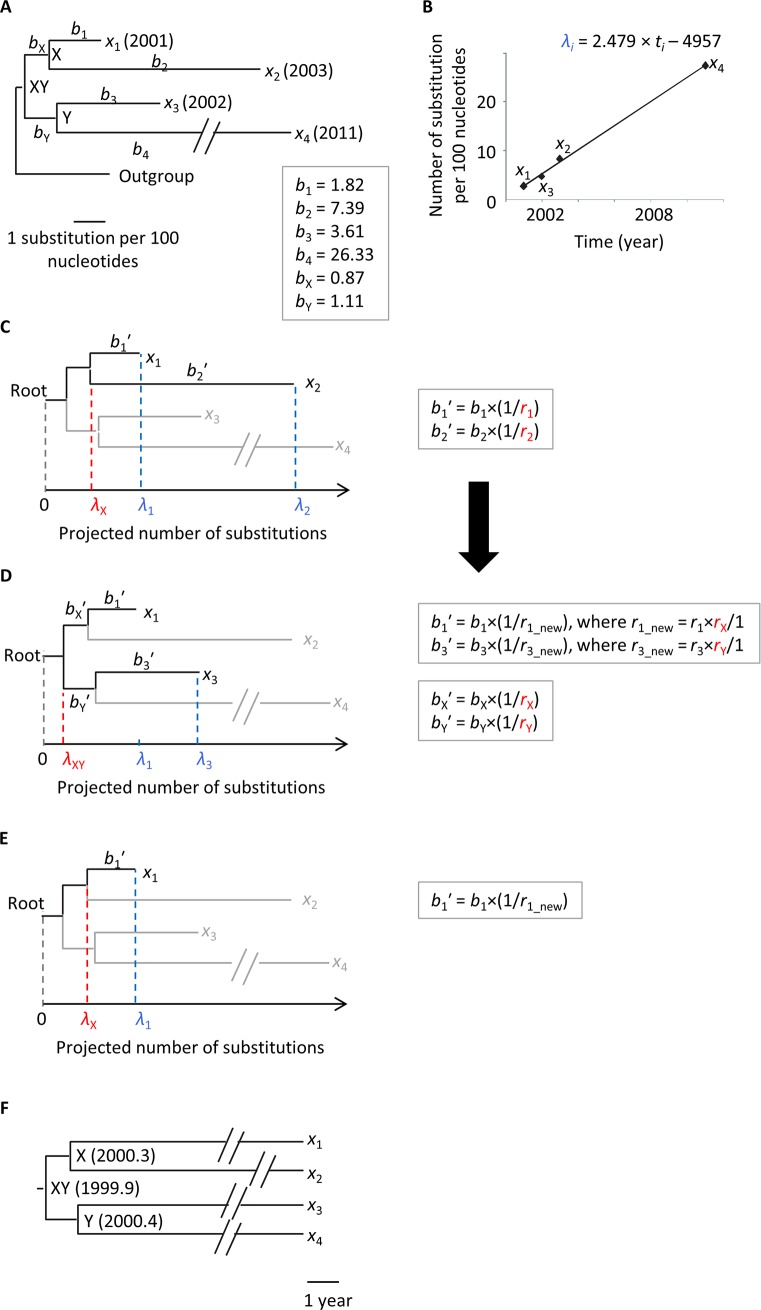
RelTime with Dated-Tips (RTDT) approach. (A) Phylogeny of five pathogen sequences (*x*_1_, *x*_2_, *x*_3_, *x*_4_, and outgroup), with branch lengths (*b*_*i*_). The year of sequence sampling (*t*_i_) is given in the parenthesis. The internal nodes are indicated by X, Y, and XY. (B) The relationship between the path lengths (*λ_i_*) from node XY to tip and sampling times (*t_i_*). For example, the point of *x*_1_ is (2001, *b*_X_ + *b*_1_). In the current example, the linear regression expression is λ_*i*_ = 2.479 × *t*_*i*_− 4957. We locate a root at the position of *λ* = 0 along the regression line. (C-E) Projected phylogeny. Root-To-Tip lengths were projected using linear regression. We first estimate relative rates at *b*_1_-*b*_4_, i.e., *r*_1_-*r*_4_ (C), and then estimate those at deeper positions of the phylogeny, i.e., *r*_X_ and *r*_Y_ (D). Lastly, we estimate the projected length from root to internal nodes, e.g., *λ*_X_ (E). (F) Estimated timetree. The final divergence times are estimated by using the regression line in panel B.

We illustrate the new approach by using a simple example dataset containing four ingroup sequences (*x*_1_, *x*_2_, *x*_3_, *x*_4_) with an outgroup sequence (**Fig 1A**) because RTDT requires a phylogeny with outgroup specified. This is different from some methods (e.g., BEAST), which jointly estimate phylogenies and divergence times without requiring the specification of outgroup sequences. In the ingroup, sequence *x*_*i*_ is assumed to be sampled in the year of *t*_*i*_ (2001, 2003, 2002, and 2011, for *x*_1_, *x*_2_, *x*_3_, and *x*_4_, respectively) and *b*_*i*_’s are the branch lengths, expressed in expected substitutions per site (**Fig 1A**). The goal is to estimate the time at internal nodes, X, Y, and XY: *t*_X_, *t*_Y_, and *t*_XY_.

This phylogeny has a time-scale measured in chronological time (*t*_*i*_) and the number of substitutions (*b*_*i*_). In the RTDT approach, we first project the path length *λ*_*i*_ (number of substitutions) from the root to a tip (*x*_*i*_) of the phylogeny under the assumption that *x*_*i*_ accumulated substitutions to the year of the sampling time, *t*_*i*_, with a constant evolutionary rate (**Fig 1B**). The projection is accomplished by first regressing the estimated length (in substitutions/site) from the ingroup latest common ancestor (XY, i.e., root) to a tip (*x*_i_) in the original tree using the corresponding sampling time. This slope is used to project root-to-tip length, *λ*_*i*_, forward in time. In our example, *λ*_*i*_ = 2.479 × *t*_*i*_− 4957, where -4957 is the intercept of the y-axis, and 2.479 is the slope. For example, the projected root-to-tip length for sequence *x*_1_ is *λ*_1_ = 2.479 × 2001–4957 = 3.48. Note that the root in this projection is an “internal-root,” which is located at the position of zero substitution along the slope (**Fig 1B**).

If the evolutionary rate were shared between branches *b*_1_ and *b*_2_, then the length from root to the internal node X, i.e., *λ*_X_, predicted by using *λ*_1_ and *b*_1_ and that predicted by using *λ*_2_ and *b*_2_ should be the same. In practice, they are not the same: *λ*_X_ is predicted to be 1.66 when using *λ*_1_ and *b*_1_ (= *λ*_1_ − *b*_1_ = 3.48 − 1.82) and 1.05 when using *λ*_2_ and *b*_2_ (= *λ*_2_ − *b*_2_ = 8.44 − 7.39), respectively. This suggests the inequality of evolutionary rates between *b*_1_ and *b*_2_. Under the RRF framework [25, 26] we, therefore, estimate their relative rates, *r*_1_ and *r*_2_, respectively, in which these two sister lineages inherited rates from their common ancestor with the minimum ancestor-descendant rate change. Assuming that the ancestral rate is equal to 1, we have the relationship, (*r*_1_ × *r*_2_)^1/2^ = 1 [25]. We used the geometric mean because relative rates could be very different from each other. We then project (recalibrate) *b*_1_ and *b*_2_ by determining the values of *r*_1_ and *r*_2,_ which reconcile the two different estimates of *λ*_x_ (**Fig 1C**).

The projected *b*_1_ is *b*_1_′ = *b*_1_ × (1/*r*_1_) and the projected *b*_2_ is *b*_2_′ = *b*_2_ × (1/*r*_2_). To determine the appropriate rate change factors, we first require that the root-to-X length (*λ*_X_) computed using *λ*_1_ and *b*_1_′, i.e., *λ*_1_ − *b*_1_′ = *λ*_1_ − *b*_1_ × (1/*r*_1_), and *λ*_X_ using *λ*_2_ and *b*_2_′, i.e., *λ*_2_ – *b*_2_ × (1/*r*_2_), be identical. Thus, we obtain the relationship, *λ*_1_ − *b*_1_ × (1/*r*_1_) = *λ*_2_ –*b*_2_ × (1/*r*_2_). Second, we use the constraint (*r*_1_ × *r*_2_)^1/2^ = 1, to solve for *r*_1_ = 0.93 and *r*_2_ = 1.08 in the current example. Similarly, for node Y, we calculate *r*_3_ and *r*_4_, which gives *r*_3_ = 0.99 and *r*_4_ = 1.01.

In the next step, we compute the relative rates of *b*_X_ and *b*_Y_, i.e., *r*_X_ and *r*_Y_, respectively. We similarly use projected branch lengths, *b*_*i*_′, and projected root-to-tip lengths, *λ*_*i*_. Here, we use the shortest root-to-tip length in each lineage of X and Y, because it is closest to a known sampling time from the root. Because *x*_1_ and *x*_3_ give the shortest length in the lineages X and Y, respectively, *λ*_XY_ on lineage X is given by *λ*_1_ – *b*_1_′ – *b*_X_′, and lineage Y gives *λ*_3_ – *b*_3_′ – *b*_Y_′ (**Fig 1D**). Thus, we seek to enforce *λ*_1_ – *b*_1_′ – *b*_X_′ = *λ*_3_ – *b*_3_′ – *b*_Y_′. Given that (*r*_X_ × *r*_Y_)^1/2^ = 1, we can calculate *r*_X_ = 1.07 and *r*_Y_ = 0.93. Note that we previously assigned *r*_X_ equal to 1, as the ancestral rate of *b*_1_ and *b*_2_ correspond to *r*_X_. Similarly, *r*_Y_ was assigned to be 1. Therefore, the relative rates in the descendant branches are rescaled. For example, the new relative rate for the branch leading *x*_1_ becomes *r*_1_new_ = *r*_1_ × *r*_X_ = 0.93 × 1.07 = 1.00. Accordingly, projected branch lengths in the descendant lineages are rescaled, e.g., *b*_1_′ = *b*_1_ × (1/*r*_1_new_).

Since all tip branch lengths are now projected, we can obtain projected lengths from root to each internal node, i.e., *λ*_X_, *λ*_Y_, and *λ*_XY_. For example, *λ*_X_ is equal to be 1.66 [= *λ*_1_ − *b*_1_′ = *λ*_1_ − *b*_1_ × (1/*r*_1_new_) = 3.48 − 1.82 × (1/1.00)] (**Fig 1E**). Using *λ*_X_, *λ*_Y_, *λ*_XY_, and the regression line, *λ*_*i*_ = 2.479 × *t_i_* – 4957 (**Fig 1B**), we obtain divergence times at the nodes XY, X, and Y to be 1999.9, 2000.3, and 2000.4, respectively (**Fig 1F**).

The dates obtained by using the above approaches are point estimates, as the underlying relative rates framework in the RelTime approach is algebraic in nature in which relative divergence times in the tree are a direct function of the branch lengths [25, 26]. Tao et al. [28] have proposed an analytical approach to estimate CIs for RelTime in which the variance contributed by site sampling and variability of rates among lineages is considered. Using that approach, RTDT produces both the point time estimate and the 95% CI of each time.

### Performance evaluation using simulated HIV data

**Fig 2:**
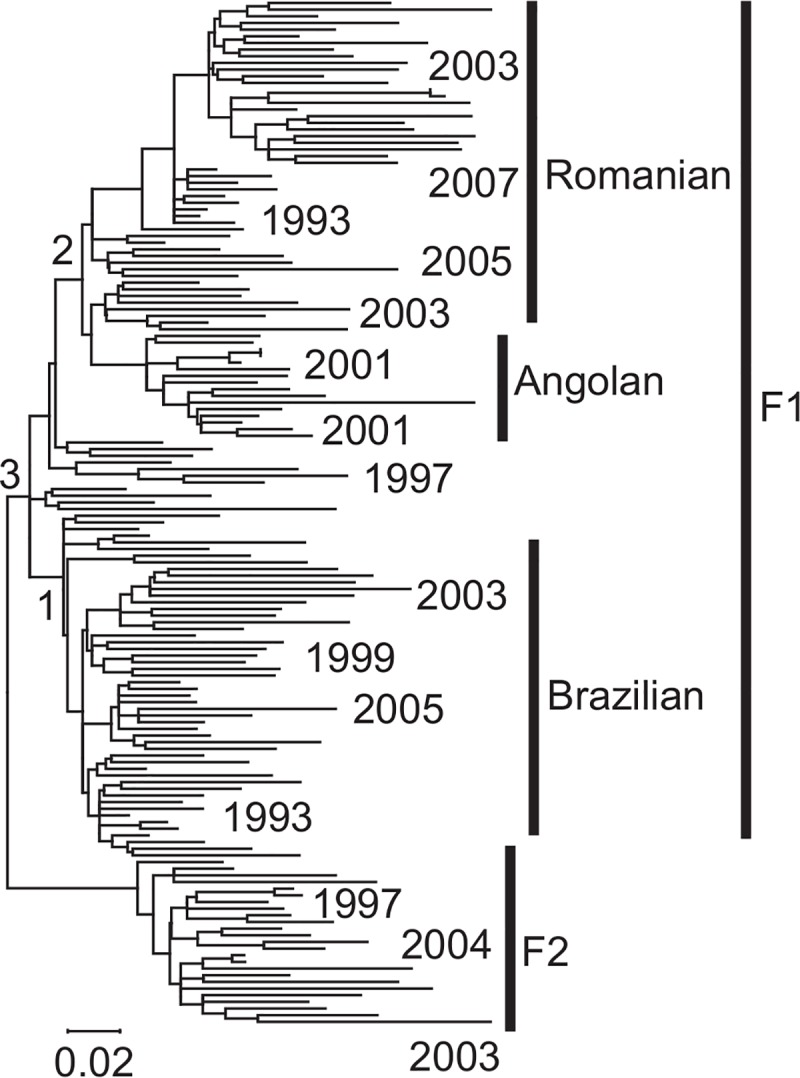
Phylogeny of HIV-1 subtype F was used as the model tree. A few sampling times are shown at the tips. The number along a node is the node ID corresponding to nodes of importance in the original study [29]; see also Table 1.

We first present results from computer simulations conducted using parameters and tree topology derived from a DNA sequence alignment of subtype F HIV-1 [29]–a representative dataset with 154 strains with various sampling times (years 1987–2007; **Fig 2**). We generated two collections of simulated datasets using this model phylogeny. In one, evolutionary rates varied independently from branch to branch (IBR model), and in the other, rates were correlated between ancestor and descendant branches (ABR model). We also generated a collection of simulated datasets in which the expected evolutionary rates were the same for all branches (constant branch rates, CBR model), to serve as the baseline model. Fifty replicates were simulated with each clock model (CBR, ABR, and IBR). To perform the analysis of RTDT, LSD, TreeTime, treedater, and BEAST, we used the correct tree topology (branching pattern) in all our analyses because we wish to compare the actual and estimated times, which would otherwise be not possible if the tree topology contained errors. Also, we did not wish to confound the impact of errors in topological inference with that of the time estimates. In the same vein, we used the correct nucleotide substitution model to keep our focus on the accuracy of the time estimates, rather than on the problems encountered by the misspecified substitution models. For each method, 50 time estimates were generated for each node in the model phylogeny.

**Fig 3:**
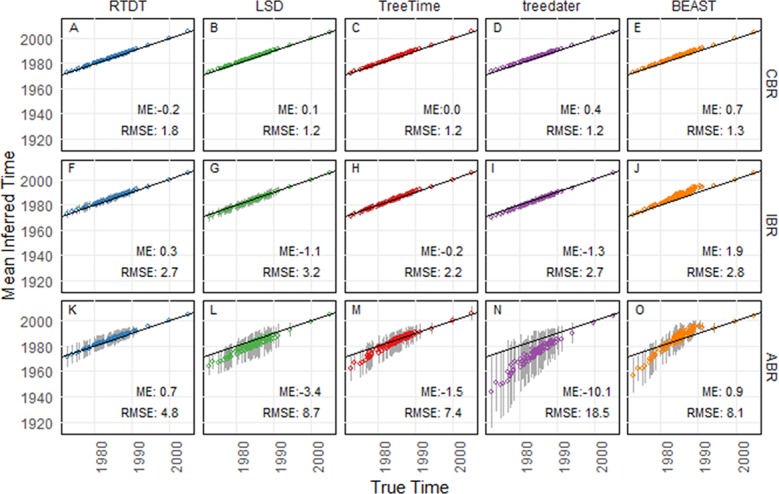
Estimates (average node time) for computer-simulated datasets of HIV-1 subtype F. The model tree is presented in **Fig 2**. RTDT (blue), LSD (green), TreeTime (red), treedater (purple), and BEAST (orange) were used for datasets simulated under the CBR clock model (A-E), IBR clock model (F-J), and ABR clock model (K-O). These averages were means from 50 simulated datasets (replicates) at each node, and error bars indicate standard deviation. For BEAST, we used a strict rate model for the analyses of datasets with CBR, and log-normal rate models were used for IBR and ABR datasets. Mean error (ME) and root mean square error (RMSE) are shown within each panel. Negative values of ME indicate overestimation, and positive values indicate a tendency to generate underestimates.

RTDT produced average time estimates that were very similar to the actual time for each node in all simulation scenarios (**Fig 3A**, **3F** and **3K**). LSD, TreeTime, and treedater also performed well for the CBR and IBR datasets (**Fig 3B–3D** and **3G–3I**). However, for the ABR datasets, average node time estimates across simulated datasets for these methods were often older than the actual times (**Fig 3L–3N**). This overestimation was more severe for deeper divergences than recent divergences, especially in the case of the treedater method (**Fig 3N**). Interestingly, even though TreeTime is a likelihood approach in which the ancestor-descendant rate shifts are penalized [19], which implies rate autocorrelation, its performance was worse than RTDT for ABR datasets.

In BEAST analyses, the use of a strict clock model for the CBR datasets resulted in excellent performance (**Fig 3E**). BEAST with the lognormal clock model also performed well for IBR databases (**Fig 3J**), even though we sampled rates from a truncated uniform distribution in IBR simulations. The use of BEAST with lognormal distribution is appropriate and effective in these analyses because the lognormal distribution fits the distribution of evolutionary rates for IBR datasets. However, BEAST did not perform well for ABR datasets (**Fig 3O**), which means its estimates produced under the assumption of evolutionary rate independence among branches are not appropriate when this assumption is violated. For ABR datasets, BEAST produced much earlier dates for deeper divergences and younger dates for more recent divergence. This result is consistent with those from a previous study where BEAST produced erroneous node times when evolutionary rates were lineage (clade) specific [30], i.e., there were local similarities in evolutionary rates.

Overall, all the methods showed similar performance for CBR and IBR datasets, but RTDT showed good results for ABR datasets as well. For ABR datasets, the average of absolute difference of estimated node time from its correct time, which is the root mean square error metric (RMSE; see **Methods** for the detail) was only five years for RTDT, while the other methods were 7–19 years for ABR datasets (**Fig 3K–3O**). Also, the estimates of the other non-Bayesian methods were systematically biased toward older times, as the average of the difference of estimates from correct times, which is the mean error metric (ME; see **Methods** for the detail), were 1.5 to 10.1 years older. For RTDT, the average was only 0.7 years younger.

**Fig 4:**
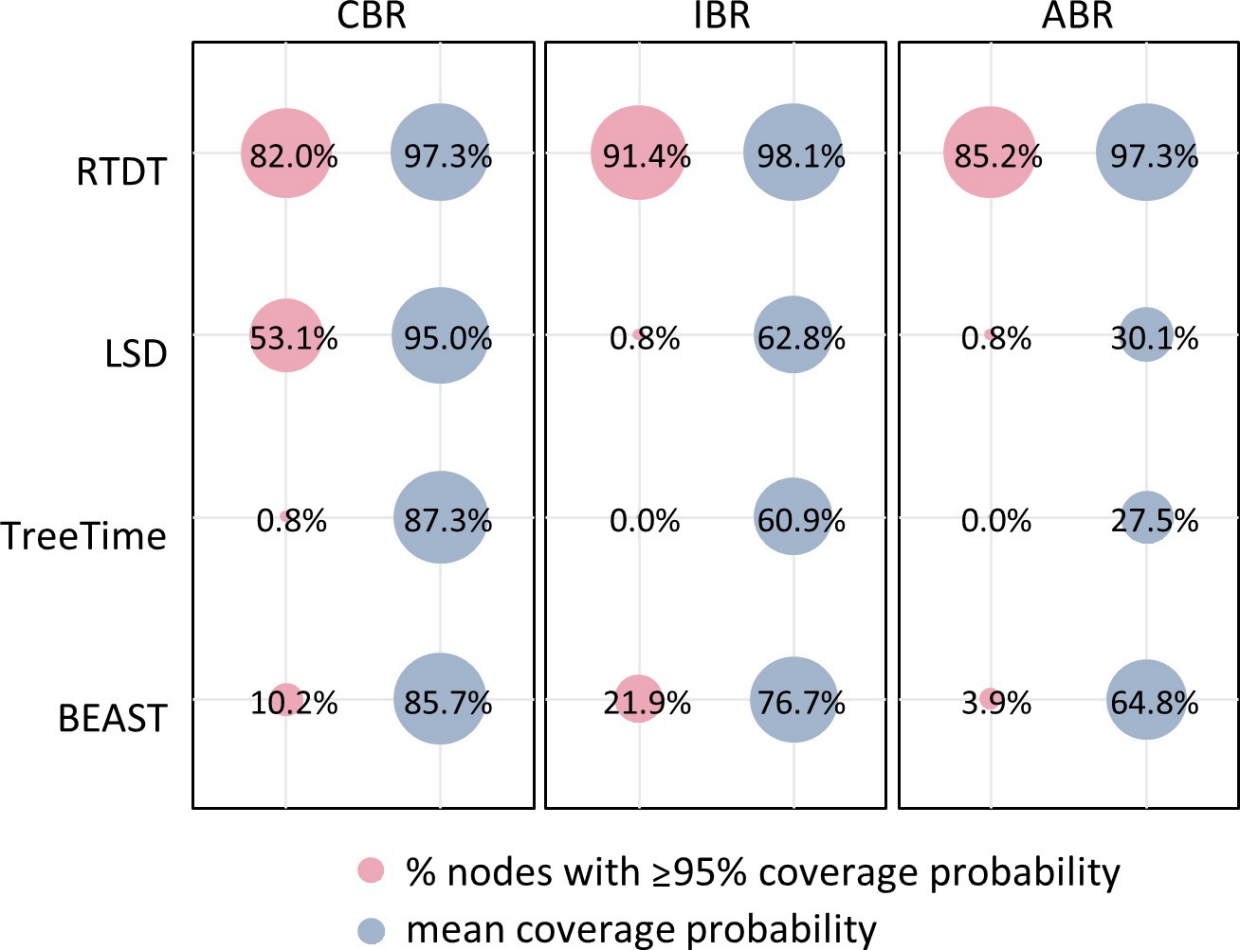
The proportion of nodes with ≥95% coverage probabilities and mean of coverage probability of CIs or HPDs for computer-simulated datasets of HIV-1 subtype F. The proportion of nodes with ≥95% coverage probability is the proportion of nodes in which ≥95% of CIs and HPDs contained the actual times, and mean coverage probability is the mean proportion of CIs and HPDs that contained the actual times across the nodes. The model tree is presented in **Fig 2**. There were 50 simulated datasets (replicates) for each of the CBR, IBR, and ABR datasets. Therefore, each node had 50 CIs or HPDs to compute the coverage probability of a node. We did not use treedater because it does not produce CIs.

Next, we evaluated the coverage probabilities, which measure how often the actual node divergence times were contained in 95% CIs or the highest posterior density intervals (HPDs) of the estimated times. The treedater method could not be included in these comparisons because it does not produce a CI for every node. The proportion of nodes with 95% coverage probabilities are shown in **Fig 4** for CBR, IBR, and ABR datasets. A vast majority of CIs produced by RTDT contained their correct times; 82% − 91% of the nodes showed ≥95% coverage probability. All other methods showed lower overall coverage probabilities, as the mean proportion of CIs that contained the actual times across the nodes was less than 77% for the datasets in which rates varied across lineages.

### Performance evaluation using simulated Influenza data

**Fig 5:**
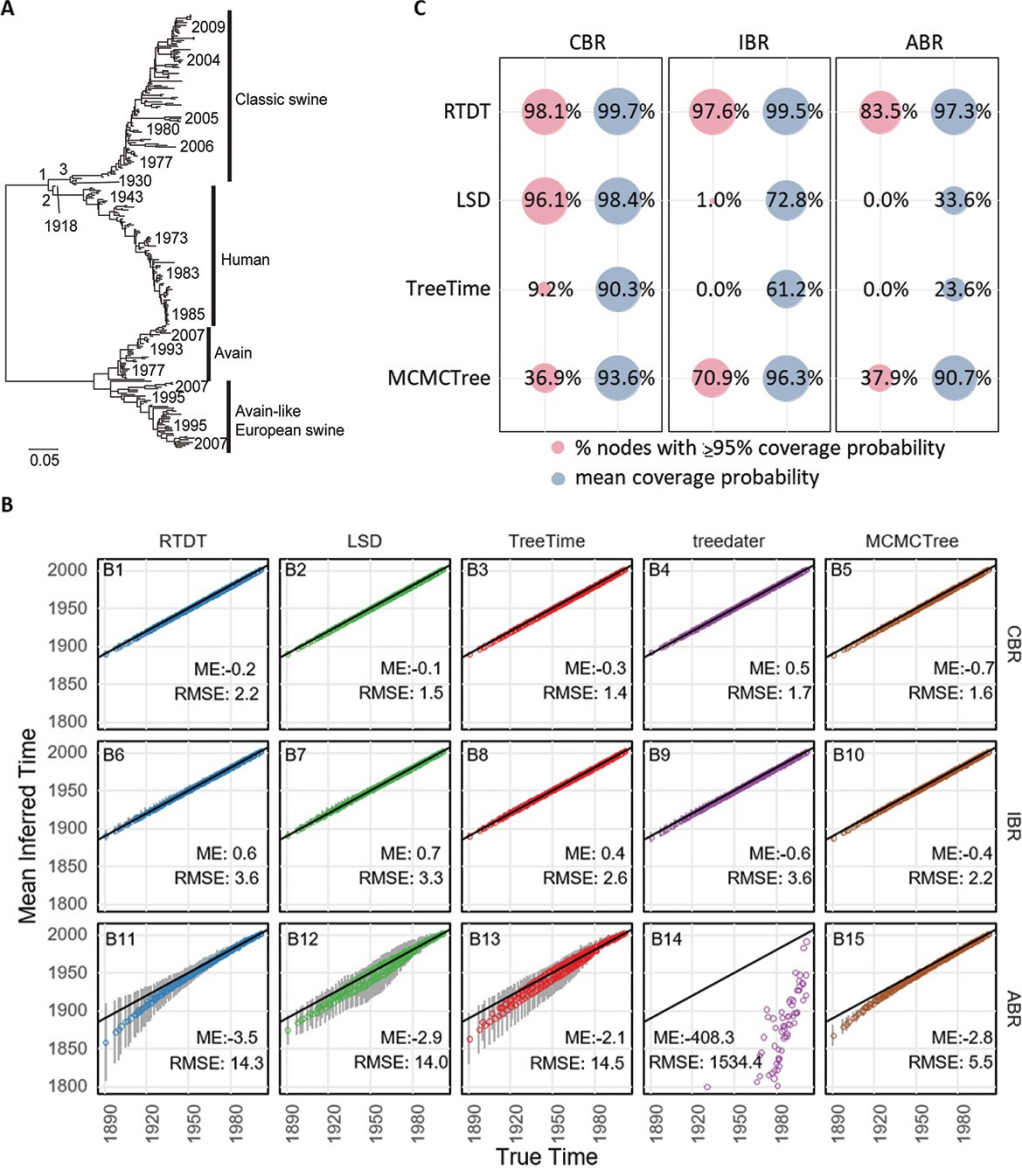
Performance of methods on the ladder-like tree. (A) Phylogeny of Influenza A strains. Sampling times are given for some tips. A number along a node is a node ID, which corresponds to those in Table 1. Fifty datasets were generated along this phylogeny with CBR, IBR or ABR. (B) Average node time estimates by RTDT (blue), LSD (green), TreeTime (red), treedater (purple), and MCMCTree (brown) for datasets with CBR, IBR, and ABR. Each time point is an average of 50 simulated datasets, and error bars indicate standard deviations. Error bars of treedater are not shown for ABR datasets, because these standard deviations were very large. MCMCTree was performed by using the correct branch rate model for each dataset. Mean error (ME) and root mean square error (RMSE) are shown within each panel. (C) The proportion of nodes with ≥95% coverage probabilities and mean of coverage probabilities of CIs or HPDs. The proportion of nodes with ≥95% coverage probability is the proportion of nodes in which ≥95% of CIs and HPDs contained the actual times, and mean coverage probability is the mean proportion of CIs and HPDs that contained the actual time across the nodes. We did not use treedater because it does not produce CIs.

We next generated datasets by using an Influenza A virus phylogeny (**Fig 5A**)[15], which contained a larger number of sequences (289 sequences) than the simulated HIV datasets. Also, this phylogeny is dramatically different from the HIV phylogeny in **Fig 2**, because of its ladder-like, highly unbalanced shape. We generated 50 datasets each under CBR, IBR, and ABR scenarios and analyzed them using RTDT, LSD, TreeTime, treedater, and MCMCTree. We used MCMCTree instead of BEAST because it was employed in the source publication [15] and because BEAST (lognormal model) required many days for each dataset to converge.

The average node time estimates of RTDT agreed well with their correct times for CBR and IBR datasets, but average node times were slightly older for deeper divergences for ABR datasets (**Fig 5B1**, **5B6** and **5B11**). Its performance was similar to or better than all other non-Bayesian methods. For Bayesian analyses, we used MCMCTree and specified the correct clock model, i.e., we used the strict, and independent, and autocorrelated clock modes for CBR, IBR, and ABR datasets, respectively. MCMCTree showed similar accuracy trends as RTDT (**Fig 5B5**, **5B10** and **5B15**), but performed better than all non-Bayesian methods for ABR datasets when considering variance among replicates for deeper node time estimates. RTDT estimates were more dispersed than MCMCTree, resulting in larger RMSE (**Fig 5B11** and **5B15**). However, CIs produced by RTDT showed very high coverage probabilities (>97%), whereas other non-Bayesian methods did not do as well (23%– 73%). MCMCTree showed intermediate performance for rate variable datasets (91%– 96%; **Fig 5C**). Therefore, RTDT is useful to generate more reliable CIs for hypothesis testing and useful especially when the dataset is very large and Bayesian methods require long computational times.

### Effect of the number of time points sampled

**Fig 6:**
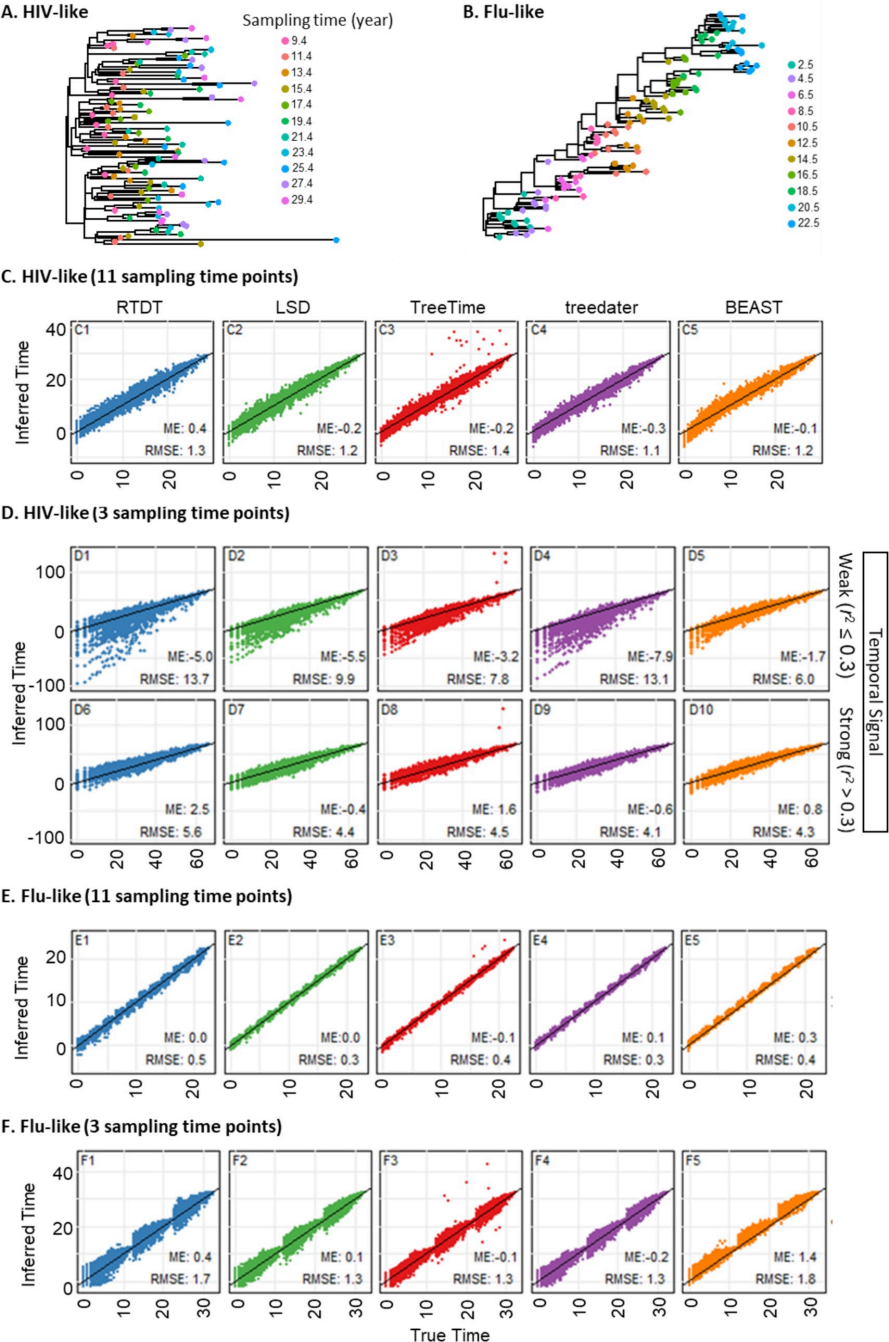
Performance of methods with a small number of sampling time points. (A and B) An example of HIV-like phylogeny (A) and Influenza-like phylogeny (B). Tips are colored based on the sampling times. In this phylogeny, the root age was set to year of 0 (actual age). Datasets were generated with independent rates. (C-F) Node time estimates by RTDT (blue), LSD (green), TreeTime (red), treedater (purple), and BEAST with log-normal rate model (orange) for datasets with eleven sampling time points (C and E for HIV-like and Flu-like phylogeny, respectively) and three sampling time points (D and F for HIV-like and Flu-like phylogeny, respectively). Mean error (ME) and root mean square error (RMSE) are shown within each panel.

We next evaluated the performance of RTDT, LSD, TreeTime, treedater, and BEAST for datasets simulated by To et al.’s [16], which mimic intra-host evolution. In these datasets, many tips shared the same sampling times (dates), and the number of distinct sampling times was only three or eleven. The sequences that were sampled at the same time may belong to different clades (HIV-like tree, e.g., **Fig 6A**) or the same clade (Flu-like tree, e.g., **Fig 6B**). Each dataset consisted of 110 sequences that were 1,000 bases long, and rates varied independently among branches (log-normal distribution of branch rates) [16]. Each simulated phylogeny was different from each other.

In the analysis of To et al.’s datasets with phylogenies similar in shape to the HIV-1 model tree (**Fig 6A**; **Fig 2**), all the methods performed well when the number of sampling time points was larger, i.e., eleven time points (**Fig 6C**). These results are consistent with those observed for the HIV-1 model tree (**Fig 3**), with the exception that TreeTime, produced much younger dates for recent divergence events for some nodes (**Fig 6C3**).

However, the performance deteriorated for all the non-Bayesian methods when only three distinct sampling times were available. They showed higher average absolute error rates than those with eleven distinct sampling time points (**Fig 6D**). We found a low correlation between sampling times and their root-to-tip lengths in these datasets (*r*^2^ < 0.3; **Fig 6D1–6D5**). Such datasets often yielded inferior results, especially for the deep nodes. BEAST also produced erroneous times when the number of sampling points was small or *r*^2^ was low, but it performed better than non-Bayesian methods (**Fig 6D5**).

For ladder-like (Flu-like) phylogenies in To et al.’s datasets (e.g., **Fig 6B**), results from eleven distinct sampling time points showed a good agreement with the actual times for all the methods (**Fig 6E**). However, the relationship showed an undulating pattern of high and low dispersion, with the low dispersions observed for nodes that were located close to the tips. For these datasets, errors of BEAST (log-normal rate model) estimates were systematically biased toward younger dates (**Fig 6E5**), more so than non-Bayesian methods. The undulating pattern of high and low dispersion, as well as the systematic error in BEAST, became more severe when the number of sampling time points was only three (**Fig 6F**). Overall, all methods showed limited accuracies on phylogenies in which the number of different sampling dates was small.

### Effects of substitution rates and sampling time intervals

**Fig 7:**
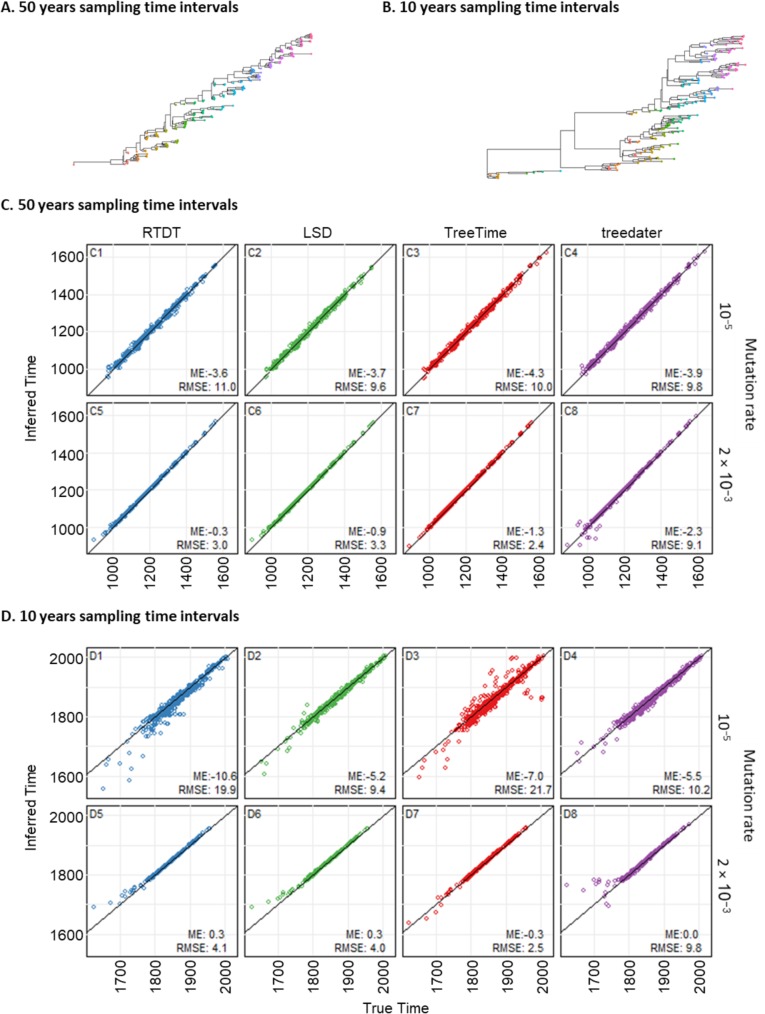
Performance of methods with various substitution rates and sampling time intervals. (A and B) Example phylogenies with sampling time intervals of 50 years (A) and ten years (B). Phylogenies with sampling time intervals of 50 years are Flu-like (A), while those with ten years were less ladder-like (B). Mutation rates in these example phylogenies are 2 × 10^−3^ substitutions per site per year. Tips are colored based on the sampling times. (C and D) Node time estimates by RTDT (blue), LSD (green), TreeTime (red), and treedater (purple) for datasets with sampling time intervals of 50 years (C) and 10 years (D), and mutation rates are slowest (10^−5^; top) or fastest (2 × 10^−3^; bottom) among the datasets. Mean error (ME) and root mean square error (RMSE) are shown within each panel. The results of the other mutations rates and those with sampling time intervals of 20 years are presented in **S1 Fig**.

We next analyzed the Sagulenko et al. [19] data, which were generated by simulating populations of size equal to 100 with evolutionary rates from 10^−5^ to 2 ×10^−3^ substitutions per site per year. Sequences were sampled every 10, 20, or 50 generations. When the sampling time interval was longer (i.e., 50 generations), those phylogenies were ladder-like (Flu-like)(**Fig 7A**). On the other hand, phylogenies with shorter sampling time intervals (10 generations) had more clades, and these shapes were still Flu-like, but less so (**Fig 7B**). Each dataset consisted of 200 sequences with 10,000 bases long.

All the methods showed an excellent performance, when the sampling time interval was larger and when evolutionary rates were faster, i.e., 50 years sampling time interval with 2 ×10^−3^ substitution rate (**Fig 7C5–7C7**), except for treedater, which sometimes produced much earlier times (**Fig 7C8**). For this sampling time interval, time estimates also agreed well when the evolutionary rate was slower (10^−5^), but these estimates were less accurate than when the evolutionary rates were faster (**Fig 7C1–7C4**), as RMSEs were 10–11 years for datasets with faster rates as compared to other datasets (2–4 years), except for treedater.

The performances tended to become worse when the sampling intervals were ten years (**Fig 7D**). Time estimates were worse for slower evolutionary rates (10^−5^), especially for RTDT and TreeTime (**Fig 7D1** and **7D3**). We found that the temporal signals for these datasets (*r*^2^ of the regression between sampling time and root-to-tip lengths) were lower than those with faster evolutionary rates as well as those with longer sampling time intervals. These results were consistent with HIV-like simulation with three sampling time points (**Fig 6D**). In addition to these issues, the performance of the treedater was abysmal for some datasets and produced much earlier dates for most of the nodes (**S1 Fig**).

### Effect of phylogenetic and sampling time uncertainties on RTDT estimates

In the above assessment, we assumed correct phylogenies and tip-sampling dates. However, some relationships in the inferred phylogenies may not be correct, and it is possible that dates for sampling times for some sequences are either unknown or can only be specified in ranges. While many available programs have provisions to deal with these uncertainties (e.g., LSD, BEAST, TreeTime, and treedater), the accuracy of times estimated is yet to be evaluated. Here we report results from our preliminary analyses to evaluate RTDT’s performance in the face of such biological realities, as an exhaustive comparative benchmarking of all the methods for many possible types and degrees of phylogenetic and sampling time uncertainties is beyond the scope of this article.

We first tested the impact of phylogenetic uncertainty on RTDT time estimates. We analyzed To et al.’s datasets for which inferred phylogenies were made available by them. 8% - 19% of the partitions in these phylogenies differed from the true phylogenies. We compared the accuracy of RTDT estimates of the time to the most recent common ancestor (TMRCA) of all the ingroup strains because it can be directly compared between the inferred and actual phylogenies when they are not the same. We found that when the number of sampling time points was large (11), the estimate of TMRCA obtained using the inferred phylogeny was excellent, as it was, on average, less than 1 year different from that obtained by using the correct tree. However, when the number of sampling time points was small (3), the performance was good for Flu-like trees (**Fig 6B;** < 1 year difference on average), but unsatisfactory for HIV-like trees (**Fig 6A;** 11 years difference on average). As noted above, RTDT tended to produce much older times for the deepest nodes, including the TMRCA, even when the correct topologies were used for HIV-like trees (**Fig 6D1**). Therefore, our limited comparisons suggest that RTDT will be useful for datasets in which the number of sampling time points is large, even if the inferred phylogeny contains errors. To et al. [16] also reported that TMRCAs estimated by LSD were not affected much by errors in inferred phylogenies.

We also tested the impact of including sequences with unknown sampling times. Sampling times for 20% of the randomly selected sequences were forgotten for IBR and ABR datasets evolved using subtype F HIV-1 phylogeny. We imputed the unknown sampling times by using a linear regression derived using the known sampling times and their root-to-tip lengths using the actual phylogenies. RTDT results with and without 20% missing sampling times were very similar (**S4 Fig**). Sagulenko et al. [19] and Votz and Frost [22] also analyzed datasets with unknown sampling times, however, their focus was to test the accuracy of imputed sampling dates and did not evaluate the impact on the divergence time estimates. Overall, our analyses suggest that a simple extension of RTDT may make it useful to include sequences with unknown or uncertain times, but this approach needs to be fully developed in the future and a comprehensive simulation analyses conducted to assess the absolute and relative efficiencies of different methods that allow for missing and uncertain sampling dates.

### Analyses of empirical datasets

We also explored some empirical datasets (**Figs 2** and **5A** and **S2 Fig** and **Table 1**) to test if RTDT was able to reproduce similar divergence times of viral strains as those reported in the original literature. We began with the HIV-1 subtype F dataset, in which we used phylogeny and other evolutionary characteristics of this dataset as a model for our HIV simulation study (**Fig 2**). We found that estimates obtained by Mehta et al. [29] were always older than those produced by using RTDT (**Table 1**). Since Mehta et al. [29] used BEAST using a lognormal rate model, this result was consistent with our simulation results, as all of these nodes are located deep in the HIV-F phylogeny (**Fig 2**), for which BEAST is expected to show a tendency to infer older dates on ABR data (**Fig 3**). Applying CorrTest [31] to this dataset, we found the autocorrelated clock model to be the best fit (*P* < 0.05). Fortunately, the difference between RTDT and BEAST dates do not contradict many of the biological scenarios presented by Mehta et al. [29], because reported (BEAST) HPDs overlapped RTDT CIs.

We next examined results for the Influenza A viral dataset, which served as a model for our influenza simulations (**Fig 5A**). Stadler and Yang [15] reported the divergence times of the most recent common ancestors of human-classical swine, human clade, and classical swine clade (**Fig 5A** and **Table 1**). They reported these divergence times with wide ranges (37–97 years) because different Bayesian methods produced different time estimates, e.g., an autocorrelated rate model in MCMCTree always produced much earlier times than the other rate models in MCMTree and BEAST (log-normal rate model). We found that RTDT estimates were very similar to BEAST with the log-normal rate model, e.g., 1813, 1898, 1910, and 1912 by MCMCTree with the autocorrelated model, independent model, BEAST (log-normal rate model) and RTDT, respectively for node 1. An ABR model fit this data set (CorrTest, *P* < 0.001), and our simulations already showed that all methods produced unreliable node time estimates for deep nodes (**Fig 5B**). Therefore, this result was also consistent with our simulation results. Nevertheless, CIs of RTDT were mainly located within the overall HPDs reported (combined HPDs of methods used in the original study).

Results from the analysis of two other HIV-1 datasets–subtypes B/D [32] and subtype D [33]–showed high concordance between RTDT and those reported in the original studies (**Table 1**). In the case of the HIV-1 subtype B/D dataset [32], phylogenies within clades for some data subsets were different. RTDT produced similar divergence times even though these trees were different, consistent with the simulation results (**S4 Fig**).

However, for the Rabies data, reported estimates were much older than RTDT (42–82 years differences), and a reported 95% HPD did not overlap the CI of RTDT. Similarly, for the HIV-2 dataset, RTDT estimates did not agree with those reported, i.e., RTDT produced node times that were much younger than those reported in the original study. Also, the reported HPDs did not overlap the CIs of RTDT. These discrepancies occurred because these data did not contain much temporal structure, as the root-to-tip lengths and sampling times did not show a good positive correlation (**S3 Fig**). Tip-dating methods are known to be adversely affected by such data, and their use is generally not recommended [34, 35].

### Computational time

**Fig 8:**
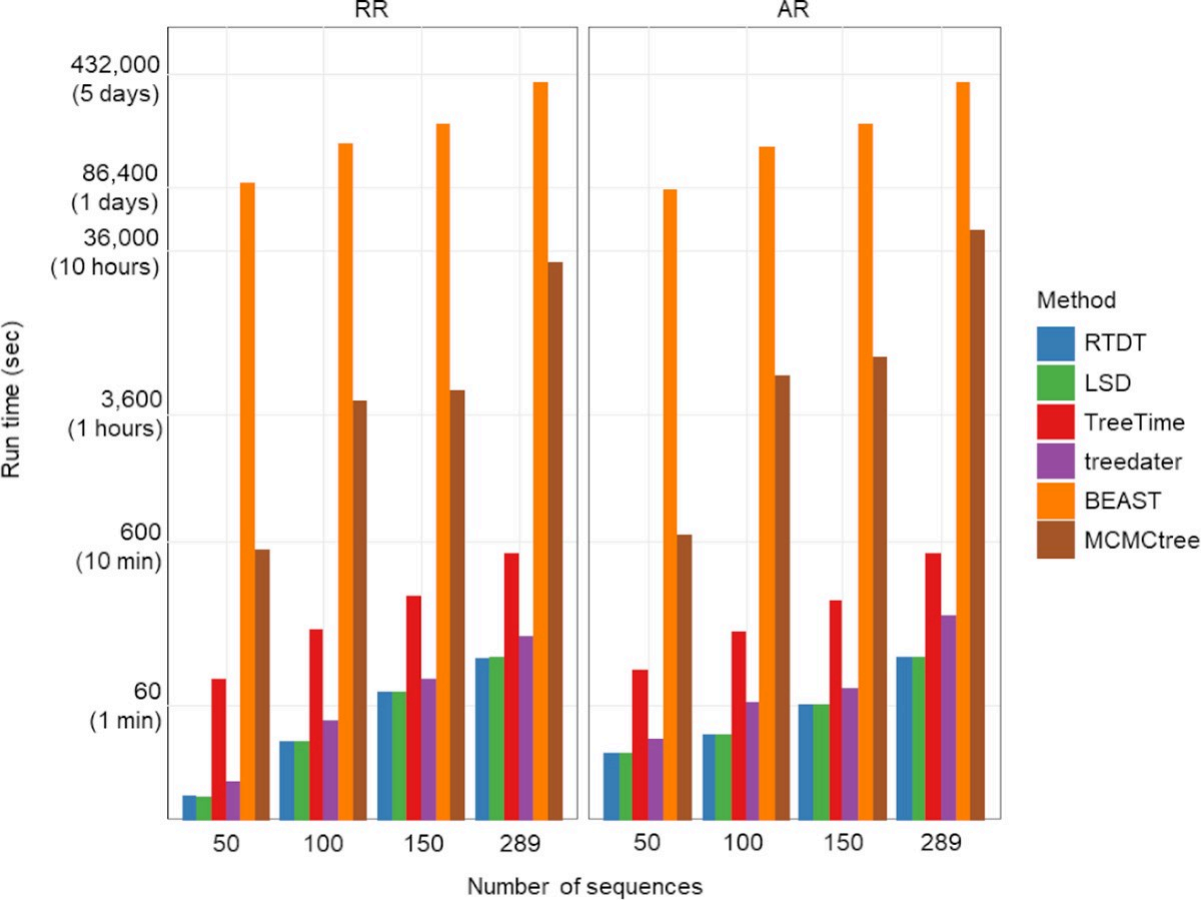
Computational time. We used simulated influenza A datasets (one IBR and one ABR datasets) that contained 289 sequences. From these datasets, we sampled 50, 100, and 150 sequences. For BEAST, we used a log-normal rate model, and correct models were selected for MCMCTree.

We also compared the computational time requirements of different methods. We did not use parallelizations and other optimizations when estimating computational efficiency to ensure a direct comparison. Nevertheless, Bayesian analyses can be performed with parallelization to reduce computational time, and non-Bayesian methods can also use parallelization when estimating branch lengths by maximum likelihood analysis. In all our analyses, we used simulated influenza A datasets (one IBR and one ABR datasets) that contained 289 sequences. From these datasets, we sampled 50, 100, and 150 sequences and ran all the analyses. As expected, all non-Bayesian methods (RTDT, LSD, TreeTime, and treedater) were much faster than the Bayesian methods (BEAST and MCMCTree). Non-Bayesian analyses completed within a few minutes, even for the largest dataset (289 sequences; **Fig 8**). However, BEAST required >24 hours for even a small dataset (50 sequences), but MCMCTree was significantly faster than BEAST. Overall, non-Bayesian methods scale well with larger datasets, and their computational time increased approximately linearly with the number of sequences and sites in a dataset.

## Discussion

We have presented a new relaxed-clock method (RTDT) to estimate times of sequence divergence using temporally sampled pathogenic strains. This new method is based on the relative rate framework in the RelTime method [25] but represents a significant advance of this framework as it removes the requirement that the sequences sampled be contemporaneous. In RTDT, there is no need to specify autocorrelation vs. independence of rates or to select a statistical distribution for branch rates, which is an advantage over Bayesian methods where such information is required *a priori*. Furthermore, RTDT requires orders of magnitude less computational time than Bayesian approaches, which makes it feasible to analyze large datasets containing thousands of sequences.

In this study, we have also provided results from our evaluation of the performance of RTDT and compared it with the performance of many other tip-dating methods using an extensive collection of simulated datasets. Based on the results from these simulations, we have developed a brief guideline for selecting methods for reconstructing pathogen timetree in empirical data analyses, which is as follows.

First, it is critical to evaluate if the dataset being analyzed contains sufficient information to estimate divergence times reliably. If the number of unique sampling time points is rather small, then the time estimates are likely to be not reliable. Also, if the sequence evolution harbors a weak temporal signal, then all the methods will tend to produce unreliable time estimates, which was evident from the difference in the performance for datasets with weak and robust temporal signal measured through the correlation (*r*^2^) between the sampling times and the root-to-tip lengths in the phylogeny. For datasets not suffering from a weak temporal signal (*r*^2^ > 0.3), RTDT may be preferred, especially when the number of sampling times is large, because it produces excellent time estimates and their CIs, and it is speedy and available in a user-friendly software (MEGA). LSD can also produce excellent time estimates, and the CIs produced are generally too narrow and may not contain correct divergence times (low coverage probabilities).

For datasets with a weak temporal signal, it is best to use Bayesian methods if they are computationally feasible. Otherwise, LSD may be applied because it is fast. In using the Bayesian method, the use of the correct clock model is important [36]. So, one should first test if the branch rates are autocorrelated by using the CorrTest [31] or Bayes factor analysis [37–39], because we found a strong signal for rate autocorrelation in HIV-1 subtype F, HIV-1 subtype D, HIV-2, and influenza datasets (**Table 1**). When the rates are found to be autocorrelated, MCMCTree with the ABR model should be used. If IBR fits the data, then MCMCTree with the IBR model or BEAST may be used. Whenever the BEAST is used, we suggest that the lognormal rate model be selected. However, users need to be aware that BEAST may produce younger dates when a tree is ladder-like. In this case, one may confirm their results by using RTDT or LSD.

The above guidelines are based on our tests in which we used the correct substitution pattern, phylogeny, and sampling dates. More advanced guidelines need to be developed through more comprehensive investigations that evaluate the robustness of all the Bayesian and non-Bayesian methods against misspecification of the substitution model and errors in estimated branch lengths, phylogenetic topologies, sampling times, and the root position. Based on the results of our preliminary analyses, we cannot recommend using sequences with missing or uncertain sampling times. Also, there is a lack of in-depth studies that have assessed the accuracies of imputed sampling times and discovered conditions under which the inclusion of sequences with missing or uncertain sampling times is genuinely beneficial, except when they are biologically required. Furthermore, in practical data analysis, it will be challenging to detect sequences with erroneous sampling times from the data itself, because a change in evolutionary rates on a lineage may leave a phylogenetic footprint similar to those caused by incorrect sampling times. Of course, one should carefully examine the relationship between sampling times and root-to-tip lengths to identify and investigate outliers, which may be affected by errors in recorded sampling times.

We also cannot recommend inferring root of the tree automatically, because of a paucity of the studies that have assessed the relative efficiencies of different methods in inferring the root and evaluated the accuracies of the estimates of root times. We have presented one example scenario (**S5 Fig**) in which the use of treedater produced a wrong root and poor time estimates. To et al. had also shown that the time estimates were less accurate when the root was inferred [16]. The challenge exists because the rates of evolution in the two branches connecting to the two descending clades of the root cannot always be de-convoluted unambiguously without an explicit outgroup. So, it is best to root the tree before molecular dating analysis.

In conclusion, the new RTDT method is expected to be useful estimating times for many datasets and their confidence intervals, because of RTDT’s computational requirements and accuracy. RTDT is implemented in the cross-platform MEGA X software (version 10.1 and later) that is freely available from http://www.megasoftware.net.

## Materials and methods

### Collection and analyses of empirical datasets

Nucleotide sequence alignments and sampling time information of nine different viruses (see **Table 1** for the detail) were obtained from the supplementary information [15], Dryad Digital Repository (https://datadryad.org/) [32], or the authors [29, 33, 40]. The HIV-1 Subtype B/D data [32] was composed of eight datasets, in which each dataset contained sequences of genes (env, gag, or pol) or the full genome with various numbers of sequences.

### Generation and collection of simulated datasets

We simulated nucleotide sequence alignments along viral timetrees obtained from the original studies (subtype F HIV-1 [29] and Influenza A [15]) and the respective nucleotide substitution rates, transition/transversion ratio, CG contents, sequence lengths, and substitution models. The nucleotide substitution rates were obtained from these original studies (3.2 × 10^−3^ and 1.7 × 10^−3^ per site per year for subtype F HIV-1 and Influenza A, respectively). The average transition/transversion ratios were 2.7 and 2.6, respectively, and the average CG contents were 38% and 41%, respectively. The nucleotide sequence lengths simulated were the same as in the original datasets (1,293 bps and 1,710 bps, respectively). The tips of branches on the timetrees were truncated according to the sampling times, which were also obtained from the original studies.

Using the Seq-Gen software [41] under the HKY substitution model [42], 50 alignments were generated for each timetree with the constant rate (CBR), randomly varying rate (IBR), and autocorrelated rate (ABR) among branches, following the methods in Tamura et al. [26]. For IBR, each mutation rate was drawn from a uniform distribution with the interval ranging from 0.5*r* to 1.5*r*, where *r* is the original mutation rate in the simulation above. For ABR, the rate variation was autocorrelated between ancestral and descendant lineages. The rate of a descendant branch was drawn from a lognormal distribution with the mean rate of the ancestral branch and the variance equal to the time duration, in which the autocorrelation parameter, *v* in Kishino et al. [43], was set to 1. Among these datasets, we removed the dataset when it included identical sequences between different taxa, because identical sequences contain no information for sequence divergence, and there is no way to know if they are sequences of the same strain or of different strain (which may become evident with longer sequences). Although the presence of real identical sequences in a dataset may be useful for population genetic analysis, e.g., coalescence and migration, but RTDT is not meant for those analyses.

In total, we used 50, 49, and 43 datasets for Subtype F HIV-1 with CBR, IBR, and ABR, respectively, and 50, 50, and 38 datasets for Influenza A virus with CBR, IBR, and ABR, respectively. Since RTDT, LSD, TreeTime, and treedater require a phylogeny with branch lengths, we employed MEGA X [44] and estimated branch lengths along correct topologies using the Maximum Likelihood (ML) method with the HKY nucleotide substitution model. These simulated datasets are available at https://github.com/cathyqqtao/RTDT, and the pipeline for the simulation is available by request.

We obtained 400 To et al. datasets (simulated alignments and estimated maximum likelihood phylogenies with correct topologies) from the LSD website [http://www.atgc-montpellier.fr/LSD/]. We excluded 77 datasets because they contained at least two identical sequences. Lastly, 240 Sagulenkoet al. datasets (simulated alignments and estimated maximum likelihood phylogenies with correct topologies) were obtained from the authors of ref. [19].

To test the impact of mistakes in the phylogeny, we obtained 400 estimated phylogenies for the To et al. datasets from the same LSD website. These phylogenies were inferred directly from the simulated sequence data by using PhyML [45].

To generate datasets with unknown sampling times, we randomly removed the sampling times of 20% of ingroup tips (i.e., 26 sampling times) from the IBR and ABR datasets simulated based on the Subtype F HIV-1 phylogeny. To perform RTDT analysis, we first imputed these unknown sampling times by using a regression line that was obtained by analyzing the relationship between available sampling times and their root-to-tip lengths. If predicted sampling time was in the future, we assigned it to be the current date.

### Analyses of simulated datasets

All RTDT analyses were conducted using MEGA X [v10.1] [44] by providing estimated ML phylogenies and correct sampling times without any uncertainties.

For LSD (v0.3) [16], TreeTime (v0.6.2) [19], and treedater (v0.3.0) [22] analysis, we provided the same sampling times and estimated ML phylogenies as used for RTDT, but these ML phylogenies contained only the ingroup sequences. Thus, we did not use the options (if any) to infer topology nor to root a tree. These methods were performed with the default parameter settings. For LSD analysis, the lower bound for the rate was 0.00001, and the parameter of variances was 1. We required divergence times between tips to be older than tip sampling times. For each dataset, CIs were computed from 100 simulated trees, in which 1,000 bps were used to generate branch lengths of simulated trees. For TreeTime analysis, we used “—confidence” option to estimate CIs. The strict clock was used for CBR data, and the relaxed clock with the default setting was used for IBR and ABR data. More specifically, for the default relaxed clock setting, we set the strength of the Gaussian priors on branch-specific rate deviation to be 1.0, and the coupling of parent and offspring rates was set to 0.5 (i.e., -relaxed 1.0 0.5). This default parameter setting represents a weak correlation. For the analysis of ABR datasets, we also tried parameter settings with stronger rate correlations, i.e., -relaxed 5.0 1.0, and parameter settings with no correlation, i.e., -relax 1.0 0, for IBR datasets. On average, the difference was < 1 year between these parameter settings. Therefore, we presented the results with the default setting. For the analysis of Sagulenkoet al. datasets, we used the inferences of TreeTime and LSD that were provided by the author of ref. [19].

The correct substitution model was used in Bayesian methods. In BEAST [v1.8.0; 14], the strict clock model was used for analyzing CBR datasets, and an independent (lognormal) branch rate model was used for analyzing IBR and ABR datasets. Correct topologies and sampling dates were provided. The constant population size model was selected for the coalescent tree prior. The number of steps that MCMC made was 100,000,000 steps, and trees were sampled every 10,000 steps for CBR datasets. For IBR and ABR datasets, we used 200,000,000 steps and sampled every 10,000 steps. To evaluate if large enough genealogies (trees) were sampled, we used the TRACER software [46] and confirmed that the number of independent information in the sampled posterior values (effective sample size; ESS) was at least 200 for most of the datasets. Among sampled trees, we excluded the first 10% of the trees as burn-in and computed the mean height of each node using the TreeAnnotator software, which is implemented in the BEAST software. To analyze To et al datasets, we used the same parameter settings as the original study, i.e., we used the input files provided at the LSD website [http://www.atgc-montpellier.fr/LSD/].

Datasets generated based on influenza A evolution were analyzed by using MCMCTree [PAML4.7; 47]. Parameter settings are the same as those in the original study [15], in which MCMCTree was used to analyze the empirical alignments. Discarding the first 20,000 iterations, 500, 2,000 and 3,000 iterations were made for CBR, IBR, and ABR datasets, respectively, and trees were sampled every 100 iterations. Strict, independent, and autocorrelated clock model was used for analyzing datasets generated with the CBR, IBR, and ABR, respectively. ESS was higher than 200 for most of the nodes for each dataset.

### Computation of average of absolute error rate and error rate

To evaluate the average of absolute error rate, we computed the root mean square error (RMSE) of each method for each simulation, following ref. [16]. RMSE = $\sqrt{\frac{1}{m\times n}\sum_{i=1}^{m}\sum_{k=1}^{n}{\left({\hat{t}}_{ik}-t_{ik}\right)}^{2}}$, where *i* is the dataset (replicate), *m* is the total number of datasets, *k* is a node, *n* is the total number of nodes, and ${\hat{t}}_{ik}$ and *t_ik_* are the estimated and true times, respectively. This measure cannot detect the direction of biases (i.e., younger or older estimates than true times), and thus, we additionally computed mean error (ME), which is the average of the signed difference between estimated node time from its true time, i.e., ME = $\frac{1}{m\times n}\sum_{i=1}^{m}\sum_{k=1}^{n}\left({\hat{t}}_{ik}-t_{ik}\right)$. ME less than zero indicates a bias towards overestimation of time because recent times in the Roman calendar have larger numerical values than earlier times, and a value greater than zero shows a tendency to underestimate time.

### Acquisition of computational time

We recorded the computational times of different methods on estimating divergence times and CIs (or HPDs) in analyses of datasets with different numbers of sequences. We subsampled 50, 100, and 150 sequences from two influenza A simulated datasets (one for IBR and one for ABR) that contained 289 sequences. For each subsampled dataset, the number of ingroup and outgroup sequences were equal to each other. For example, a subset of 50 sequences contained 25 ingroup sequences and 25 outgroup sequences. For RTDT, we recorded the computational time of inferring divergence times and CIs with the option of using molecular sequences. For LSD and TreeTime, we recorded the sum of computational times for inferring the ML tree and for computing the divergence times and CIs. This computational time represents the total runtime of LSD and TreeTime analyses for a given molecular alignment. For treedater, we first recorded the sum of computational times for inferring the ML tree and for computing the divergence times. Then we multiplied this runtime by 50 to represent the total runtime of analyzing 50 bootstrap replicates to get CI of a root node in treedater. For MCMCTree, we used the same chain length as the analysis of the Influenza A simulation. For BEAST with a log-normal rate model, we used 300,000,000 chains to ensure the convergence for the dataset with the largest number of sequences. All analyses were conducted on a single core without parallelization on the Linux machine with 896 GB RAM.

## Supporting information

S1 Fig Performance of methods with various substitution rates and sampling time intervals (Extension of Fig 7). Each point is a node time estimate, and the colors indicate mutation rates to generate datasets.
(TIF)

S2 Fig Phylogenies from the published literature for empirical datasets. Phylogenies of HIV-1 subtype B/D (A), HIV-1 subtype D (B), HIV-2 (C), and rabies (D) are shown. Branch lengths were the number of substitutions. Sampling times were indicated for a few sequences. A number along a node is a node ID, which corresponds to that in **Table 1**. Those node times were reported in the original study. Phylogenies of HIV-1 subtype F and Influenza A are presented in **Fig 2** and **Fig 5A**, respectively.
(TIF)

S3 Fig Relationships of root-to-tip lengths and sampling times for empirical data. The empirical data was listed in **Table 1**.
(TIF)

S4 Fig Impact of incorrect sampling times. Each dataset contained incorrect sampling times of 20% of ingroup tips. RTDT was performed by using these incorrect sampling times with correct phylogenies. The average node times across datasets agreed very well with their true times for both IBR and ABR datasets (A and B, respectively), and these accuracies were similar to when we provided correct sampling times (**Fig 3**).
(TIF)

S5 Fig The prediction of the root position and divergence time. (A) The true timetree, where R is the root of interest. Sequences were simulated based on the true timetree under an IBR model for the HIV data. (B) The ML phylogeny for this dataset was correct, except that the position of the root was not available when the outgroup sequence was excluded from the data, and it was better to use an outgroup (panel C). The treedater program predicted a wrong root and time (1971 rather than 1982) for the dataset that excluded the outgroup sequence (panel D). The use of outgroup resulted in a better time estimated (panel E). This means that lengths of two branches (*b*_*x*_ and *b*_*y*_) emanating from node R could not be determined reliably without the availability of the outgroup sequence.
(TIF)
