# Supplementary figures and images for: A new method for inferring timetrees from temporally sampled molecular sequences
Source: PLoS Comput Biol. 2020 Jan 17;16(1):e1007046. doi: 10.1371/journal.pcbi.1007046 (PMC7018096; doi:10.1371/journal.pcbi.1007046)

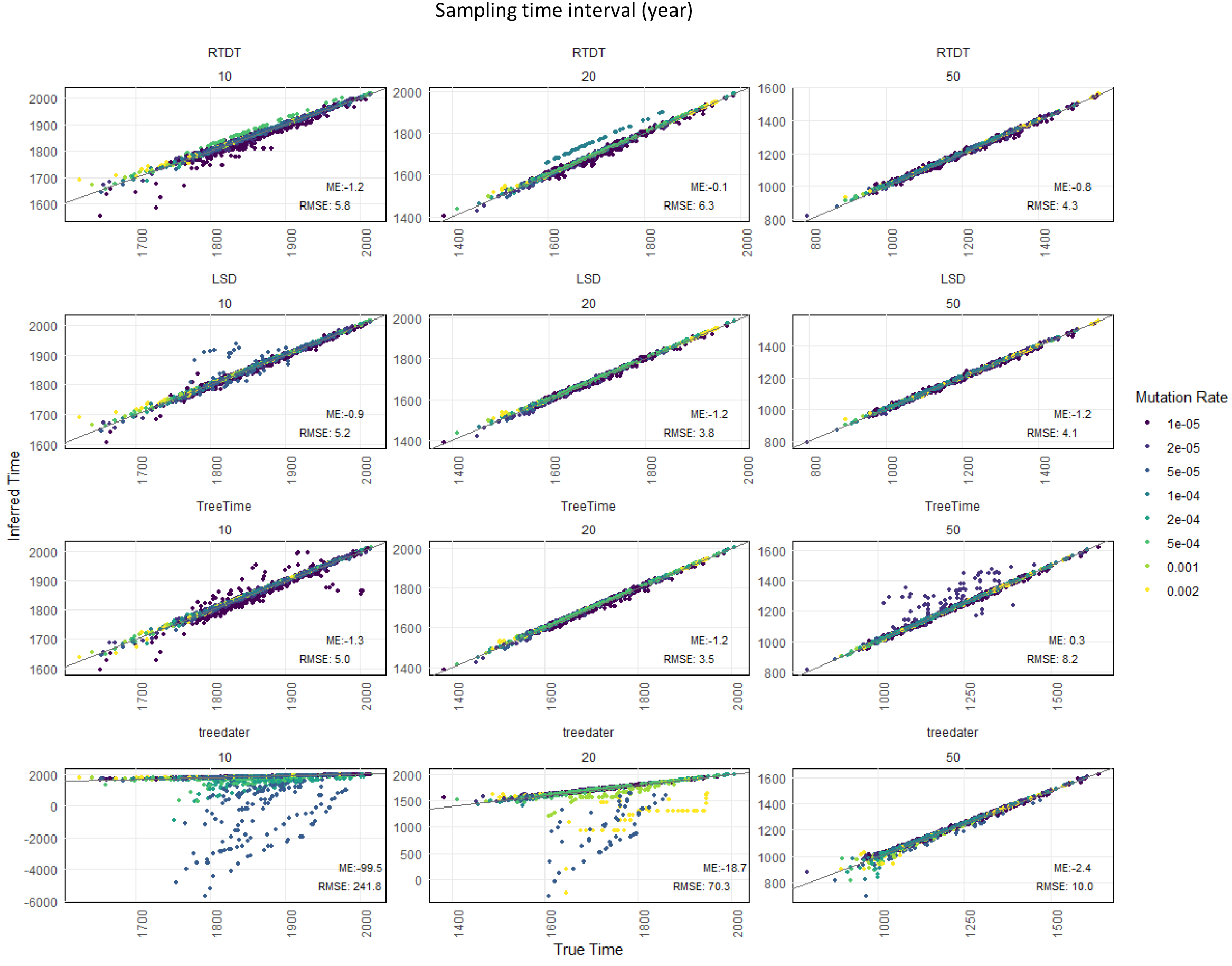

Supplement: S1 Fig — Each point is a node time estimate, and the colors indicate mutation rates to generate datasets. (TIF) [file pcbi.1007046.s001.tif]

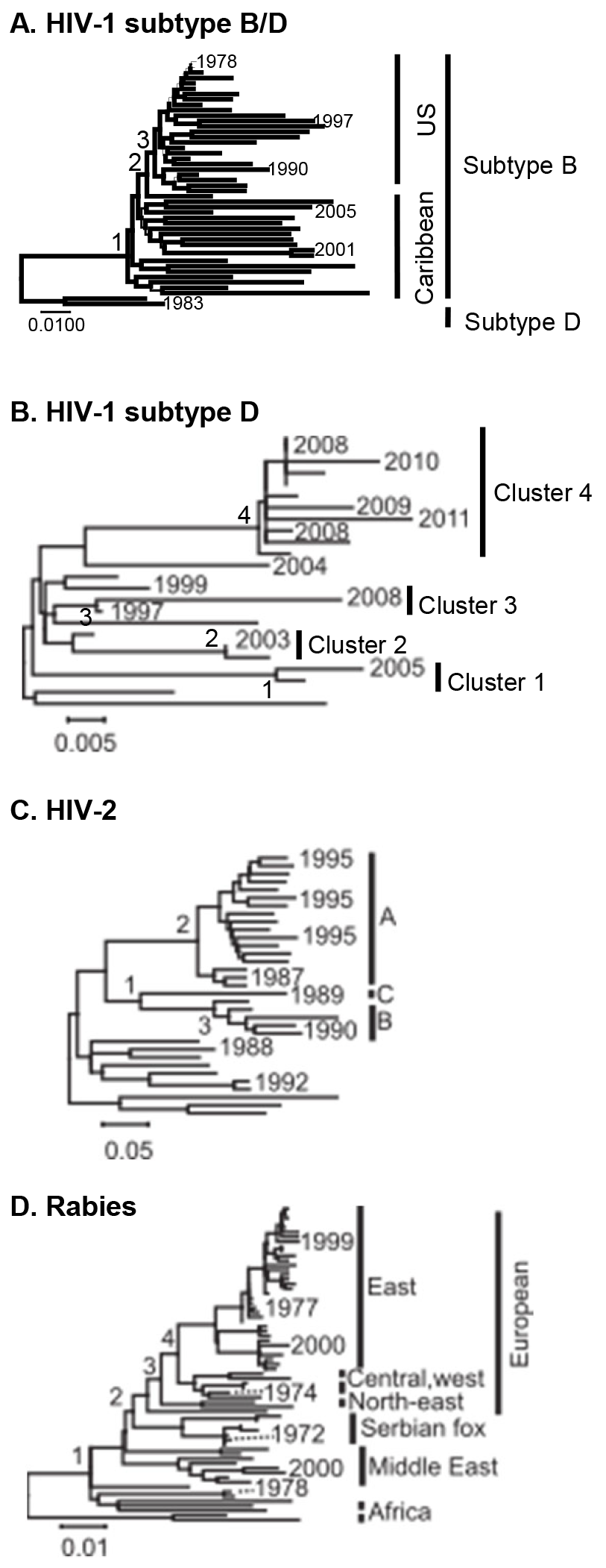

Supplement: S2 Fig — Phylogenies of HIV-1 subtype B/D (A), HIV-1 subtype D (B), HIV-2 (C), and rabies (D) are shown. Branch lengths were the number of substitutions. Sampling times were indicated for a few sequences. A number along a node is a node ID, which corresponds to that in Table 1. Those node times were reported in the original study. Phylogenies of HIV-1 subtype F and Influenza A are presented in Fig 2 and Fig 5A, respectively. (TIF) [file pcbi.1007046.s002.tif]

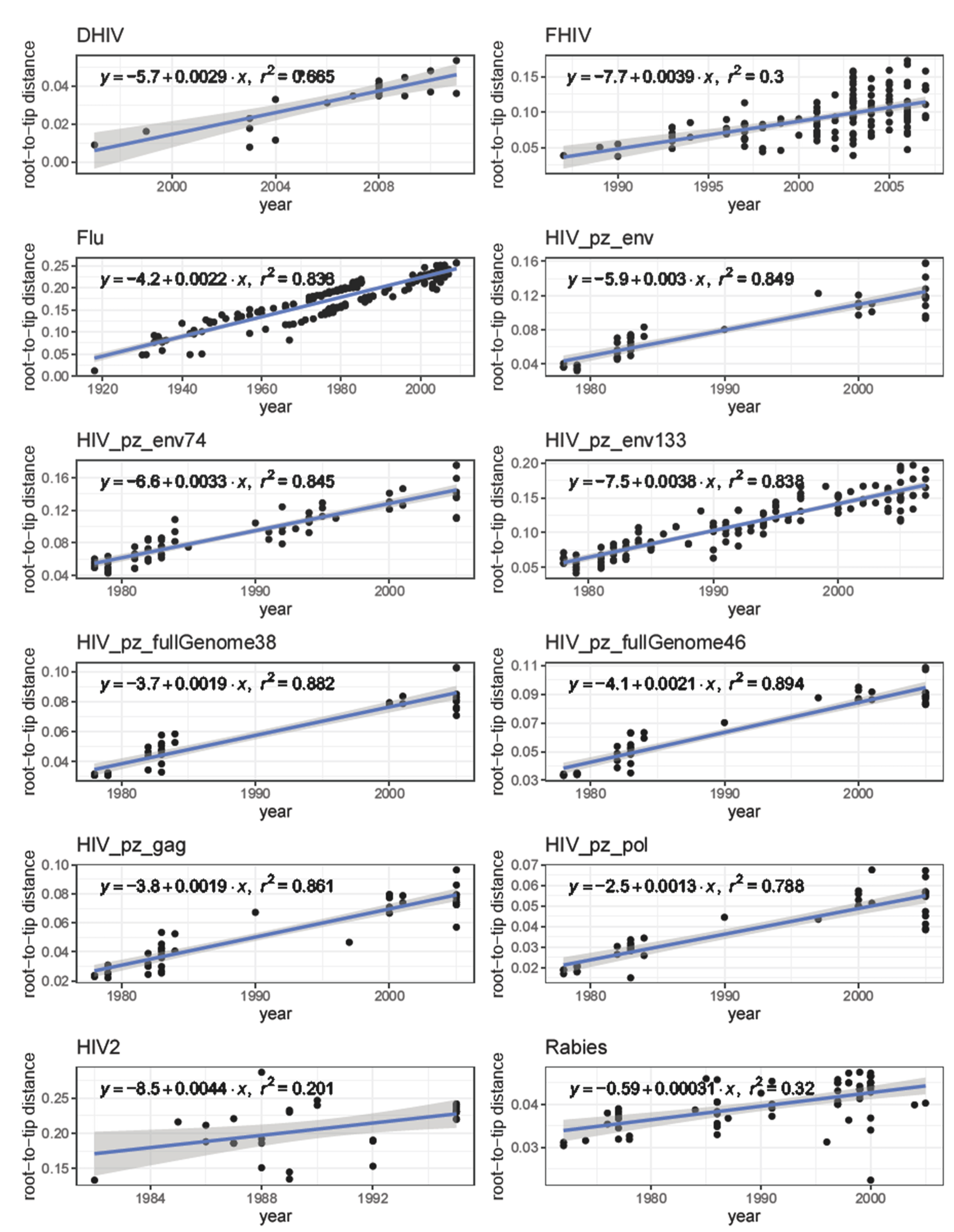

Supplement: S3 Fig — The empirical data was listed in Table 1. (TIF) [file pcbi.1007046.s003.tif]

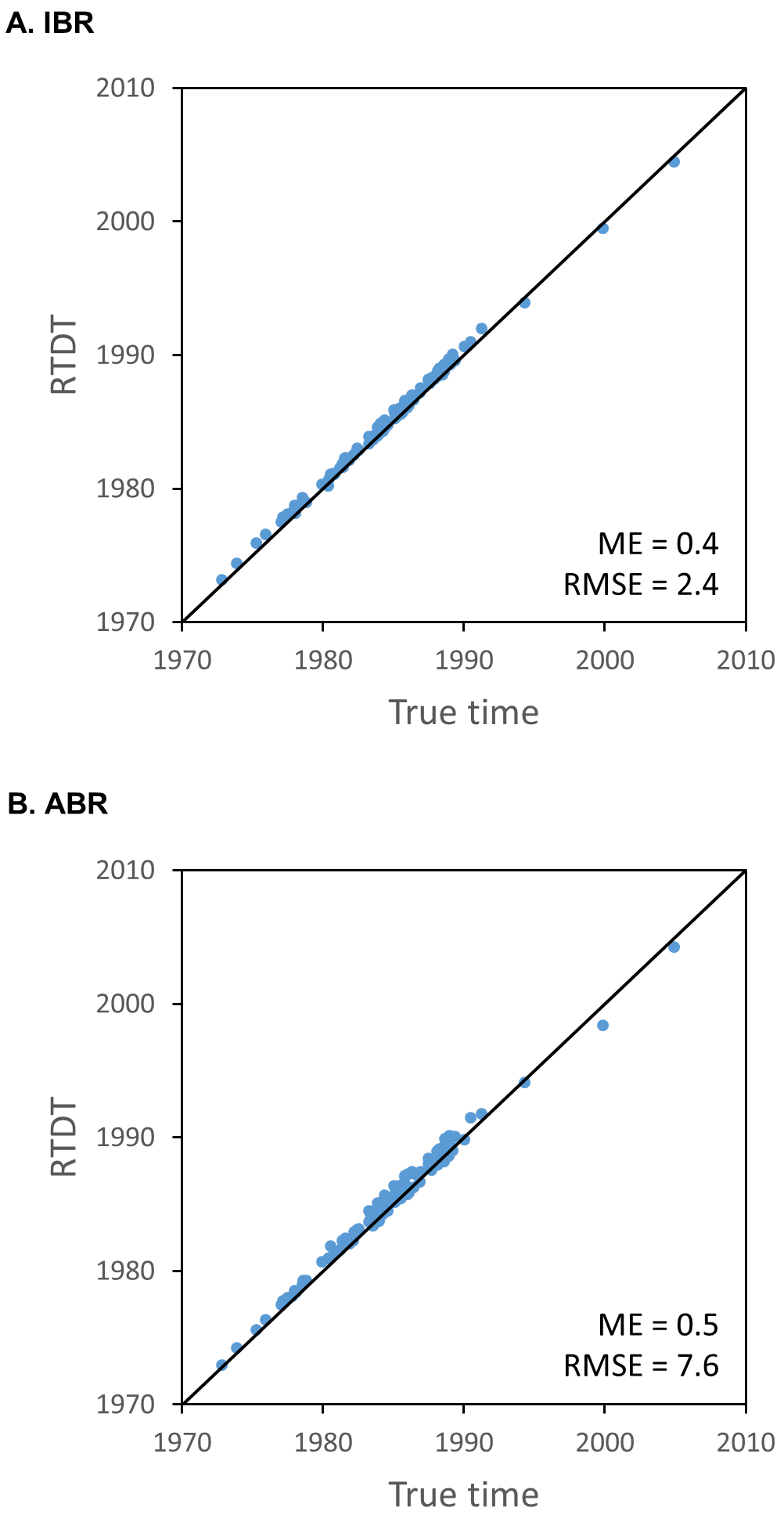

Supplement: S4 Fig — Each dataset contained incorrect sampling times of 20% of ingroup tips. RTDT was performed by using these incorrect sampling times with correct phylogenies. The average node times across datasets agreed very well with their true times for both IBR and ABR datasets (A and B, respectively), and these accuracies were similar to when we provided correct sampling times (Fig 3). (TIF) [file pcbi.1007046.s004.tif]

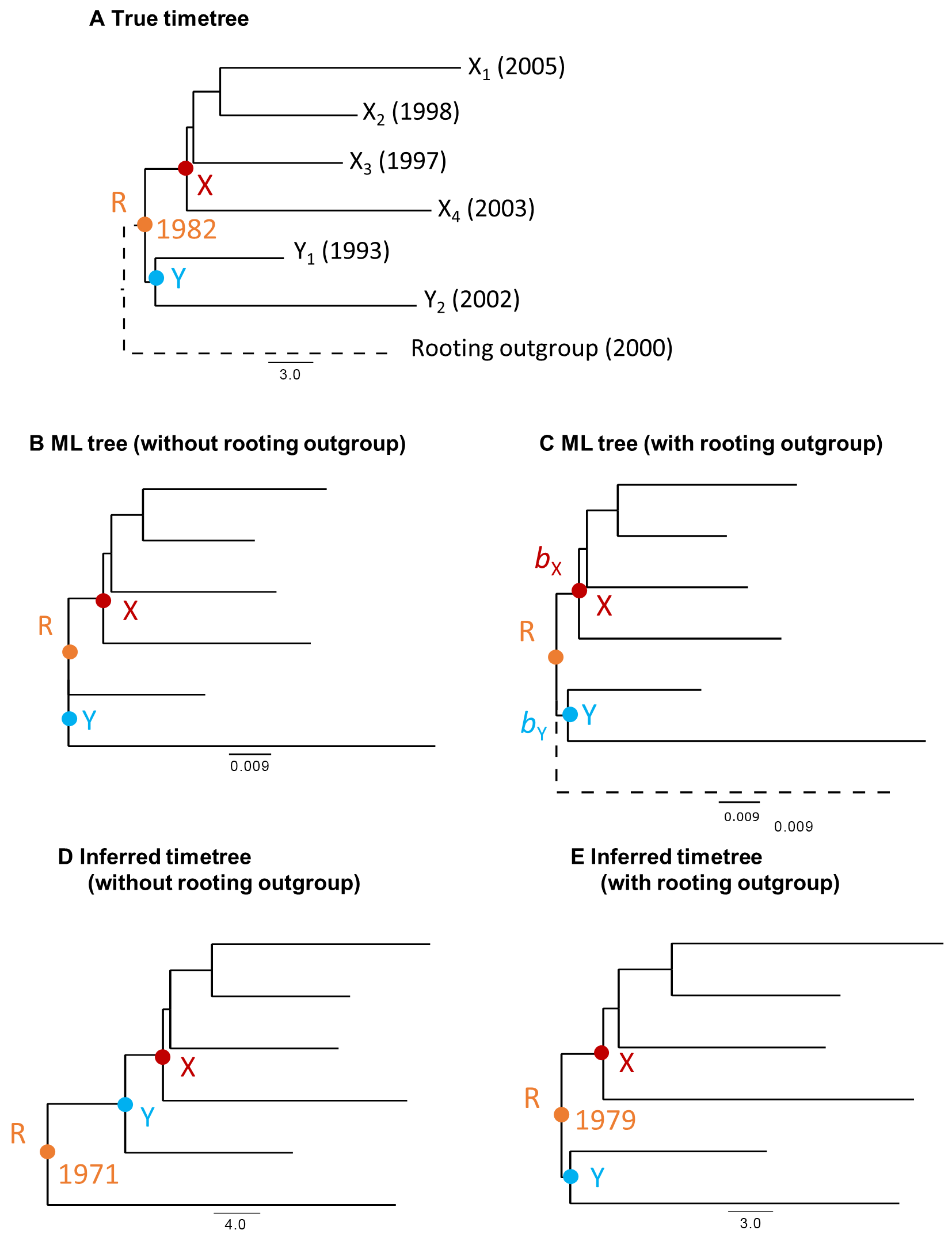

Supplement: S5 Fig — (A) The true timetree, where R is the root of interest. Sequences were simulated based on the true timetree under an IBR model for the HIV data. (B) The ML phylogeny for this dataset was correct, except that the position of the root was not available when the outgroup sequence was excluded from the data, and it was better to use an outgroup (panel C). The treedater program predicted a wrong root and time (1971 rather than 1982) for the dataset that excluded the outgroup sequence (panel D). The use of outgroup resulted in a better time estimated (panel E). This means that lengths of two branches (bx and by) emanating from node R could not be determined reliably without the availability of the outgroup sequence. (TIF) [file pcbi.1007046.s005.TIF]
